# Selection and Modelling of a New Single-Domain Intrabody Against TDP-43

**DOI:** 10.3389/fmolb.2021.773234

**Published:** 2022-02-14

**Authors:** Martina Gilodi, Simonetta Lisi, Erika F. Dudás, Marco Fantini, Rita Puglisi, Alexandra Louka, Paolo Marcatili, Antonino Cattaneo, Annalisa Pastore

**Affiliations:** ^1^ Department of Molecular Medicine, University of Pavia, Pavia, Italy; ^2^ Dementia Research Institute at King’s College London, The Wohl Institute, London, United Kingdom; ^3^ Bio@SNS Laboratory, Scuola Normale Superiore, Piazza dei Cavalieri, Pisa, Italy; ^4^ Department of Bioinformatics, Technical University of Denmark, Kongens Lyngby, Denmark

**Keywords:** antibody selection, hypervariable loops, intrabodies, modelling, misfolding proteins, ALS

## Abstract

Amyotrophic lateral sclerosis (ALS) is a neurodegenerative disorder associated to deteriorating motor and cognitive functions, and short survival. The disease is caused by neuronal death which results in progressive muscle wasting and weakness, ultimately leading to lethal respiratory failure. The misbehaviour of a specific protein, TDP-43, which aggregates and becomes toxic in ALS patient’s neurons, is supposed to be one of the causes. TDP-43 is a DNA/RNA-binding protein involved in several functions related to nucleic acid metabolism. Sequestration of TDP-43 aggregates is a possible therapeutic strategy that could alleviate or block pathology. Here, we describe the selection and characterization of a new intracellular antibody (intrabody) against TDP-43 from a llama nanobody library. The structure of the selected intrabody was predicted *in silico* and the model was used to suggest mutations that enabled to improve its expression yield, facilitating its experimental validation. We showed how coupling experimental methodologies with *in silico* design may allow us to obtain an antibody able to recognize the RNA binding regions of TDP-43. Our findings illustrate a strategy for the mitigation of TDP-43 proteinopathy in ALS and provide a potential new tool for diagnostics.

## Introduction

Amyotrophic lateral sclerosis (ALS) and Frontotemporal dementia (FTD) are distinct but genetically correlated fatal neurodegenerative diseases. ALS is characterized by the selective degeneration of motor neurons that typically appears in middle-aged patients (average age 55 years) and progresses to muscle atrophy followed by complete paralysis. Death is caused by respiratory failure and typically intervenes within 3–5 years from diagnosis. The disease is predominantly (90%) sporadic, but familial cases (fALS) are found in ca. 10% of the cases (Prasad et al., 2019). FTD is also a midlife-onset disease that is clinically heterogeneous and characterized by changes in behaviour, personality, and/or speech (Mackenzie and Neumann, 2016). Because of a remarkable overlap in manifestations, the two diseases are now considered a disease continuum, with 50% of ALS patients presenting cognitive impairment (15–20% recognized as FTD), and 15% of FTD patients having motor impairments (Devenney et al., 2015; Burrell et al., 2016).

Several proteins have been implicated in these diseases. Among them is the TAR DNA-binding protein 43 (TDP-43), a DNA/RNA-binding protein ubiquitously expressed, and predominantly localized in the nucleus (Ayala et al., 2008; Prasad et al., 2019). TDP-43 is a modular protein that is involved in different aspects of RNA metabolism including transcription, splicing, transport, and scaffolding (Buratti and Baralle, 2008; Cohen et al., 2011; Liu et al., 2017). The architecture of TDP-43 comprises a partially folded N-terminal domain, two RNA-binding RRM tandem domains (RRM1 and 2), and an unstructured C-terminus that contains a so-called prion-like motif (Buratti and Baralle, 2001; Winton et al., 2008; Lukavsky et al., 2013; Mompeán et al., 2016). An hallmark of the TDP-43 related pathologies is the mislocalization, accumulation and consequent aberrant aggregation of TDP-43 in the cytoplasm where the protein is heavily post-translationally modified (Suk and Rousseaux, 2020). TDP-43 aggregates are also associated to other diseases, such as Alzheimer’s disease (AD), Parkinson’s disease (PD), and Huntington’s disease (HD) (Buratti and Baralle, 2009; Gao et al., 2018).

Clinical mutations of TDP-43 are rare and seem to occur mainly, but not exclusively, in the C-terminus of the protein (Pesiridis et al., 2009; Barmada et al., 2010). This observation had originally suggested that this region is the main cause of protein aggregation and misfolding. More recently TDP-43 fragments containing only the RRM domains or the whole region from the N-terminus to the end of RRM2 have been demonstrated to aggregate and misfold also in the absence of the C-terminus (Budini et al., 2015; Chen et al., 2019; Zacco et al., 2019) indicating that TDP-43 contains multiple aggregation-prone hotspots. Accordingly, clinically relevant mutations occurring in the two RRM domains have been described (Chen et al., 2019).

Despite the advancements made in understanding TDP-43 aggregation, too many details of the mechanism remain unclear. Lack of information partially arises from a lack of adequate research tools able to accurately probe aggregation. In this regard, antibodies constitute a ductile means widely used in research and in clinics, thanks to their high binding affinity and specificity. Antibody applications extend from quantitative *in vitro* measurements to *in vivo* studies. When expressed as intrabodies inside cells (Biocca et al., 1990; Cattaneo and Chirichella, 2019), they can for instance be used to sequester protein aggregates reducing cell toxicity (Meli et al., 2014). They are also great assets in diagnostics and basic science as they may be used in super-resolution microscopy, allowing visualization of protein aggregates at the nanoscale as in the recently developed DNA-PAINT methodology (Schermelleh et al., 2019; Sograte-Idrissi et al., 2019; Oi et al., 2020).

Among the natural antibody scaffolds, variable domains of the heavy chain antibody (VHHs) (also named nanobodies) offer specific advantages over normal antibodies but also respect to single chain Fv (scFv) fragments (Bird et al., 1988) or domain antibodies (dAbs) (Ward et al., 1989) or other antibody mimetics. Natural VHHs were first identified in camelids (Saerens et al., 2005) which are typically single variable heavy chain domains of ca. 110 amino acids that are derived from heavy-chain-only antibodies (V_H_), devoid of the light chain partners. A major advantage of camelid VHHs, with respect to immunoglobulin-derived dAbs (24), is their ability to specifically recognize antigens with affinities similar to those obtained by whole antibodies despite their smaller size, and the absence of the hydrophobic VH-VL interface. VHHs are also usually more stable, with melting temperatures as high as 90°C, and higher resilience to detergents and denaturants. Given their small size, good tissue penetration, and low immunogenicity, VHHs have been developed for different neurodegenerative disorders such as AD, Lewy body disease, PD, and HD, and in the attempt to block or prevent aggregation (Harmsen and De Haard, 2007; Khodabakhsh et al., 2018; Hoey et al., 2019; Messer and Butler, 2020).

Here, we describe a new naïve library of llama VHHs, and exploit it to select directly from TDP-43 cDNA a new anti-TDP-43 VHH, which we named VHH5. Usually, VHH libraries are obtained from immunized animals, and are used in different display platforms (phage, yeast, and ribosomal, etc.), that require the immunizing protein for antibody detection from the library. We constructed instead a *llama glaba* naïve VHH library in the SPLINT (Single Pot Library of Intracellular Antibodies) format in yeast, followed by antibody selection with the two-hybrid-based Intracellular Antibody Capture Technology (IACT) (Visintin et al., 1999; Visintin et al., 2002; Visintin et al., 2004). This approach allows direct selection of antibodies from antigen cDNA, with no need to express and purify the protein antigen (Meli et al., 2009). Based on the amino acid sequence deducted from the DNA sequence of the selected VHH5 intrabody, we performed an *in silico* prediction of the antibody structure. The resulting model was used to suggest mutations that optimized the expression of VHH5 in bacterial cells, enabling the experimental biochemical validation of the intrabody. We demonstrate that structure prediction is a powerful tool to guide carefully planned mutagenesis that can facilitate soluble intrabody production. To the best of our knowledge, this is the first detailed description of an anti-TDP-43 intrabody. This new VHH opens new avenues for diagnostic, to interfere with protein aggregation and for imaging applications by super-resolution microscopy (Messer and Joshi, 2013; Schermelleh et al., 2019).

## Materials and Methods

### Llama Glaba VHH Library Construction

Naïve blood samples (40 ml) from two non immunized female llamas were kindly provided by the Biopark Zoom (Cumiana, Turin, and Italy) which is an approved public husbandry Zoo, which operates under the following law: legislative decree 21 March 2005, n. 73 (Gazzetta Ufficiale n. 100, 2 May 2005). The blood samples were taken from the two llama animals as part of the normal periodic blood testing of these animals. Periferal blood lymphocytes were separated by Ficoll-Histopaque-1077 (Sigma-Aldrich) discontinuous gradient centrifugation followed by washing with the phosphate buffered saline (PBS) solution and stored at −70°C. Total RNA was isolated from 10^7^ leucocytes by acid guanidinium thiocyanate-phenol chloroform extraction (using TRIzol RNA Isolation Reagents, Thermo Fisher Scientific). RNA integrity was assayed by agarose gel electrophoresis. The total RNA (5 µg) was retrotranscribed in cDNA using the Reverse Transcriptase Core Kit (Eurogentec RT-RTCK-03), with the following thermocycles: 25°C for 10 min, 48°C for 30 min, 95°C for 5 min. The VHH sequences were amplified from cDNA using previously described primers (van der Linden et al., 2000). We used a degenerate forward primer (VH1-Back BssHII**)** annealing to the hinge region of each heavy chain-only IgG isotype corresponding to the amino acid sequence (E/Q/K/*)V (Q/K)LQ (E/Q)SG), with the BssHII restriction site (underlined) VH1-Back BssHII: GC GCG CAT GCC VAG GTS MAR YTR GTN SAG TCW GG and two reverse primers Lam-07 NheI and Lam-08 NheI that respectively anneal the llama long-hinge heavy chain antibody (cIgG2), and the short-hinge antibody (cIgG3) (Hamers-Casterman et al., 1993) with the NheI restriction site (underlined) Lam-07 NheI: GCTAGC GGA GCT GGG GTC TTC GCT GTG GTG CG; Lam-08 NheI GCTAGC TGG TTG TGG TTT TGG TGT CTT GGG TT.

The PCR protocol consisted of an initial denaturation step at 98°C for 1 min followed by 10 cycles of 98°C for 10 s, 55°C for 30 s, and 72°C for 30 s, followed by 10 cycles of 98°C for 10 s, 60°C for 30 s, and 72°C for 30 s, followed by 10 cycles of 98°C for 10 s, 65°C for 30 s, and 72°C for 30 s, and a final extension step at 72°C for 3 min. The resulting unique ∼450 bp PCR fragment was purified from 1.5% highly pure agarose gel using the Wizard® SV Gel and the PCR Clean-Up System (Promega), digested with BssHII and NheI (New England Biolabs), re-purified and ligated (T4 DNA Ligase, NewEngland Biolabs) into BssHII, and NheI digested pLinker220 IACT plasmid (Visintin et al., 2004). This plasmid carries the LEU2 gene, involved in the synthesis of Leucine (L), the 2 μm origin of replication for transformation in yeast, and the selection marker (Ampicillin) and the origin of replication (ColE1 ori) for selection in bacteria. Ligation of the library (∼1 μg) was transformed by electroporation into Max Efficiency *E. coli* DH5α cells (Invitrogen). Transformation efficiency was estimated by plating serial dilution aliquots on Luria Broth (LB)/ampicillin (100 μg/ml) agar plates, incubated overnight at 37°C, and assessed by colony count. ∼1 million cells were inoculated the next day into 1 l of LB, Sea Prep Agar and ampicillin for library amplification (Elsaesser and Paysan, 2004). An aliquot of the inoculated mixture was plated on LB/ampicillin (100 μg/ml) agar plates to determine the effective colony count. The inoculated Sea Prep Agar was then poured in a pre-chilled sterile stainless-steel container (∼200 × 300 × 50 mm^3^; Neolab, Heidelberg, and Germany) on wet ice in a cold room and left on ice at 4°C for 1.5 h, and transferred to an incubator at 37°C for 40 h. The visible spherical bacterial colonies embedded in the semi-liquid gel were collected by centrifugation at 8,000 *g* for 20 min at room temperature. The pellet was washed with 100 ml of LB medium and centrifuged again at 8,000 g for 20 min at room temperature. Plasmid DNA from the pellet was extracted using a Qiagen GIGAprep kit, following the manufacturer’s instructions.

### NGS Llama Library Sequencing

The obtained llama library was sequenced as previously described (Fantini et al., 2017). To attach sequencing adapters to the VHH sequences, a ligation-based approach was designed. DNA adapters were synthesized harbouring overhangs complementary to the cleavage product of the restriction enzymes BssHII and NheI, used for excising the scFv fragment from the plasmid. The forward and reverse strands of the adapters were synthesized independently and annealed *in vitro* (1:1 ratio, 95°C 5 min, and 95→25°C in 5°C steps 1 min/step). Before annealing the reverse strand was phosphorylated (0.2 nmol of oligos, 10U PNK (NEB) at 37°C for 1 h, and at 65°C for 20 min) to allow ligation. The VHHs were excised from the library plasmid (∼2 μg of the library were digested for 3 h at 37°C with 4U of NheI (NEB), and for 3 h at 50°C with 4U of BssHII (NEB)) and ligated to the adapters (forward adapter:VHH:reverse adapter in 10:1:10 ratio, ∼200 ng library 400 U T4 ligase (NEB), and overnight at 16°C). Ligation was run on an agarose gel and the band corresponding to the single insert with the 5′ and 3′ adapters was resolved and purified with the MinElute Gel Extraction Kit (Qiagen).

The library was quantified by Qubit dsDNA HS Assay Kit (ThermoFisher Scientific), diluted to 4 nM, and denatured with 0.1 N NaOH (5 min at room temperature), neutralized and diluted again in buffer HT-1 (Illumina) to a final concentration of 12.5 pM. Equimolar denatured Phi-X Control V3 DNA (Illumina) was spiked-in 20% volume as an internal quality control and to increase the sample diversity according to Illumina guidelines. Sequencing was performed on the MiSeq system with the Reagent Kit v3 (Illumina), using 350 and 250 cycles for the forward,1 and reverse reads respectively.

Raw data were demultiplexed from. bcl files into separate. fastq files with bcl2fastq-1.8.4 (Illumina), using the following barcodes as indexes: i1 = TCAGCG, i2 = GATCAC, i3 = CTGAGA, and i4 = AGCTTT. To take into account the different lengths of shifter sequences introduced with the sequencing adapters, a specific number of nucleotides was discarded from the start of the reads (R1 index i1 = 0, i2 = 1, i3 = 7, and i4 = 8; R2 index i1 = 13, i2 = 12, i3 = 11, and i4 = 10). Reads were purged from adapter dimers, quality-filtered (Phred Score 32), and trimmed in sequences of the same length (R1: 320bp; R2: 220bp) with trimmomatic-0.32 (Bolger et al., 2014). All the sequences whose forward and reverse reads both survived from the previous step were selected, taking advantage of the Perl script fastq-remove-orphans.pl, and which is part of the fastq-factory suite (https://github.com/phe-bioinformatics/fastq-factory). The VHH nanobody library reads were merged using PEAR (van der Linden et al., 2000), a pair-end read merger available at http://sco.h-its.org/exelixis/web/software/pear/.

### Intrabody Selection

The TDP-43 gene (residues 1–414) was cloned in pMicBD1 plasmid (pMicBD1-TDP-43 bait plasmid) and transformed in L40 yeast. The strain was grown in 1% Yeast Extract, 2% Bacto Peptone, 2% Glucose, and at pH 5.8 to an OD_600_ of 0.6. Cells were washed in 1xTE (10 mM Tris, 1 mM EDTA, and pH 7.5), and resuspended in 0.5 ml of 1xTE/1xLiAC (10 mM Tris, 1 mM EDTA, and 0.1 M Lithium acetate dehydrate pH 7.5). Cells (100 µl) were added to 100 µg of salmon tested DNA (STD) and 200 ng of pMicBD1-TDP-43 plasmid with 600 µl of 50% PEG/1xTE/1xLiAC (40% (w/v) PEG 4000, 10 mM Tris-HCl, 1 mM EDTA, and 0.1 M lithium acetate dehydrate pH 7.5) and spun at 150 rpm for 30 min at 30°C. DMSO (70 µl) was added and the cells were heat shocked at 42°C for 15 min, put in ice for 2 min, centrifuged, resuspended in 100 µl of 1 × TE and plated on Synthetic Designed liquid minimal medium lacking tryptophan (SD-W) plates.

For IACT screening, the strain expressing the LexA-TDP-43 bait was grown overnight at 30°C in SD-W media. The overnight culture was diluted in 1 l of pre-warmed rich medium YPAD (1% Yeast Extract, 2% Bacto Peptone, 0.01% Adenine, 2% Glucose, and pH 5.8) and cultured from OD_600_ 0.3–0.6. Cells were centrifuged, washed in 150 ml of 1 × TE, and resuspended in 15 ml of 1 × TE/1 × LiAC. Salmon tested DNA (STD) (10 mg), and the VHH llama DNA library (250 µg) cloned in the pLinker220 prey plasmid were added. The mixture was transferred in a flask with 140 ml of 50% PEG/1xTE/1xLiAC and incubated at 150 rpm for 30 min at 30°C. DMSO (17.6 ml) was added and the cells were heat shocked at 42°C for 15 min under gentle mixing. The flask was then put in ice for 5 min and the cells were washed three times with YPA (1% Yeast Extract, 2% Bacto Peptone, 0.01% Adenine, and at pH 5.8), and recovered in 1 l of YPAD for 1 h at 30°C. A quarter of the cells were washed three times with SD-WHL (SD without, Tryptophan, Histidine, and Leucine), resuspended in 5 ml of SD-WHL, and plated on SD-WHL Petri dishes. The remaining cells were washed in SD-WL (same of SD-WHL but with 0.05% Histidine), resuspended in 200 ml SD-WL and grown overnight at 30°C. The next morning the cells were washed and resuspended in SD-WHL, plated on SD-WHL Petri dishes, and incubated at 30°C for 4–5 days. Ninety nine clones were picked and re-streaked onto a SD-WHL and SD-WL plates. A liquid β-galactosidase (β-gal) assay, adapted from (Möckli and Auerbach, 2004), was performed using a 96-well plate. A small amount of the biomass from single colonies was resuspended in 50 µl of lysis buffer (20 mM Tris HCL pH 7.5, 333 U/ml lyticase) and incubated for 2 h at 37°C. 50 µl of a solution made of 60 mM Na_2_HPO_4_, 40 mM NaH_2_PO_4_, 10 mM KCl, 1 mM MgSO_4_, pH 7.0, X-gal at 20 mg/ml (170 µl), and β-mercaptoethanol (30 µl), was added to each well and incubated for 2 h at 37°C. Strong prey–bait interactions were identified by the development of blue color.

### Colony PCR and Fingerprint Analysis

Colony PCR and fingerprint analysis were performed only on double positive colonies (His+/LacZ+). The clones were lysed using 10 µl of buffer (20 mM Tris HCl pH 7.5, 300 U/ml lyticase). The VHH of each clone was amplified by PCR using primers located at the 5′ and 3′ of the VHH in the pLinker220 plasmid. The primers were pL220 Fw (5′-AAG CTT ATT TAG GTG ACA CTA TAG-3′) and pL220 Rev (5′- CTT CTT CTT GGG TGC CAT G-3′). The PCR reaction was performed as follows: 3 min at 95°C, followed by 30 cycles at 95°C for 30 s, 50°C for 30 s and 72 °C for 40 s, 5 min at 72°C, and then 4°C to store. The PCR mixture (8 µl/20 µl) was digested with the restriction enzymes NlaIV and AluI, for 2 h at 37°C, to identify a specific pattern for each isolated VHH. Digested fragments were resolved using 8% polyacrylamide gel electrophoresis, followed by ethidium bromide staining. Once the different patterns were highlighted, six individual clones were selected to extract the prey DNA from yeast. Each plasmid was transformed by electroporation, using DH5α Emax cells into bacteria to obtain a pure and monoclonal preparation.

### *In vivo* Epitope Mapping of the anti-TDP-43 VHH5

To characterize the epitope recognized by the anti-TDP-43 VHH5 the original LexA-TDP-43 bait was truncated in two fragments named LexA-N-term + RRM1-2 (residues 1–258) and LexA-C-term (residues 259–414) and transformed in L40 yeast as described above*.* These strains were then transformed with the pLinker220 plasmid carrying the VHH5 with the same protocol and plating the cells on SD-WL or SD-WHL. To further narrow down the region carrying the epitope a second cycle was done, splitting the region found positive (1–258) into four smaller baits, the N-terminus (1–105), RRM1 (106–176), RRM2 (192–258), and a fragment of RRMs (160–208) which contains the linker between RRM1, and 2 (not to be confused with RRM1-2 which is represents a construct comprising the tandem domains). The anti-TDP-43 VHH5 was transformed in L40 yeast strains individually carrying one of the smaller baits.

### Initial Model Generation

The most suitable template was identified by submitting the sequence of the target protein to the BLAST search (https://blast.ncbi.nlm.nih.gov/Blast.cgi) against the PDB database. Models were built both by the SWISS-MODEL (Waterhouse et al., 2018) and the ABodyBuilder (Leem et al., 2016) servers. The semi-automated procedure was used in SWISS-MODEL where alignment between the template and the target was fed manually.

### Loop Generation

Modelling of the complementarity-determining region (CDR) H3 loop was carried out using the Sphinx algorithm (Marks et al., 2017). The input to Sphinx is a protein structure or a model (in PDB format) and the location and sequence of the loop to be modelled. We used the best SWISS-MODEL structure to model the loop region comprising residues 94–114. Once a complete set of decoys was generated, a statistical potential was used to reduce the set to only 500 structures, which were then scored using SOAP-Loop (Dong et al., 2013) to produce a ranking. SOAP-Loop was assessed by the average global root mean square deviation (RMSD) of the top ranked model for each loop. From the ranking that was generated based on the frequency of how often similar conformations were selected and the energy of single conformations, we selected ten models for the loop which we used as a mould to perform the docking between the nanobody and TDP-43.

### Model Refinement

Molecular dynamics (MD) simulations were performed using the NAMD 2.13 package (Phillips et al., 2020) with the CHARMM36m force field. Input files were generated by CHARMM-GUI (Jo et al., 2008; Lee et al., 2016). The structures were solvated with the TIP3P water model in a rectangular box such that the minimum distance to the edge of the box was 10 Å under periodic boundary conditions. An appropriate number of Cl^−^ counterions were added to neutralize the protein charge. The time step was set to 2 fs throughout the simulations. A cutoff distance of 12 Å for Coulomb and van der Waals interactions was used. Long-range electrostatics was evaluated through the Particle Mesh Ewald method. The two energetically best models—one provided by the SWISS-MODEL server homology modelling pipeline and one by the ABodyBuilder antibody modelling pipeline—were refined by energy minimization. 20,000 steps of conjugated gradient energy minimization were carried out 1) without constraints, 2) with positional constraints on the backbone heavy atoms of residues 1–70 and 77–135, and 3) with positional constraints on all heavy atoms of residues 1–70 and 77–135. Throughout these minimizations—providing replicas 1, 2, and 3 for each model—the applied force constant was 1.0 kcal mol^−1^Å^−2^. The energy minimization resulted in six models that after additional 10,000 steps of energy minimization were subjected to 1 ns of equilibration at 303.15 K and 1 atm. The production runs (100 ns) were performed under the same conditions except that all positional constraints were removed. A similar procedure was adopted on the energetically best model obtained after the H3 loop generation as ranked according to SOAP-Loop ranking. The model was subjected to 10,000 steps of energy minimization and 1 ns of equilibrations at 303.15 K and 1 atm. This was followed by an 80 ns production run.

Trajectories were visualized and analysed with the VMD program (Humphrey et al., 1996). Every tenth frame of each trajectory was loaded, for a total of 500 structures. Structural alignment was achieved on the whole molecule for the ABodyBuilder structures and on the region 1–121 for the SWISS-MODEL structures. Coordinates were extracted with a stride value of 10, resulting in 50 structures, and visualized in PyMOL.

### ClusPro

Antigen-antibody binding was carried out based on the NMR structure of human TDP-43 tandem RRM1-2 in a complex with a UG-rich RNA (PDB code 4bs2) from which the RNA molecule was removed. Molecular docking was performed by using the ClusPro software (Kozakov et al., 2017). The standard inputs of ClusPro are two PDB files, one denoted as the ligand, and the other one as the receptor. To influence docking, an attractive force was set on the residues of H3 using default parameters. The calculations were repeated on each of the ten best structures obtained by Sphinx. Cluster selection was made to exclude solutions that did not show any contact between the CDR loops and the TDP-43 ligand. An additional filtering step was included to remove all the solutions in which less than ten CDR residues were involved in molecular interactions with the antigen. A residue was defined as interacting if any of its atoms was at less than 4 Å distance from any antigen atom. Similarly, each solution was annotated based on the number of contacts with the first, and second domain in the TDP-43 structure. All the representative structures from then ten ClusPro runs were then pooled together and analysed to identify conserved interaction patterns with the antigen. The interface RMSD (iRMSD) between each pair of solutions was then computed, by superimposing the antigen structure, and measuring the RMSD of the Cα atoms in the CDR regions of the respective interacting antibody. Clustering of the solution was then performed on the complete distance matrix, by using the DBScan algorithm from the Python package SciKit-Learn (https://scikit-learn.org/stable/modules/generated/sklearn.cluster.DBSCAN.html), using the parameters eps = 9, and min_clust = 3 (Pedregosa et al., 2011). The clustering results were then visualised by transforming the distance matrix to a two-dimensional space using the t-SNE algorithm in SciKit-Learn (Van Der Maaten and Hinton, 2008) (https://scikit-learn.org/stable/auto_examples/index.html). The models were visualised by the Pymol software.

### Sequence Analysis

AGGRESCAN (Conchillo-Solé et al., 2007) was used to predict the aggregation properties of VHH5. The standard input for AGGRESCAN is the polypeptide sequence(s) consistent with FASTA format. In the output, the regions of the sequence with the highest predicted aggregation propensity are highlighted in red in the peptide sequence column and appear as peaks in the profile graphs. The position of the CDR loops was obtained by the http://cao.labshare.cn/AbRSA/abrsa.php server (Li et al., 2019).

### VHH5 Production

Preliminary attempts to produce the protein in *E. coli* were done using a pET-17b which encoded a fusion protein with an N-terminal PelB leader sequence and a C-terminal (His)_7_-tag. Since this strategy proved unsuccessful, VHH5 was recloned by PCR into a pET-SUMO plasmid, and expressed in BL21 (DE3) pLysS cells as a fusion protein with an N-terminal SUMO solubilization domain and a (His)_6_-tag. Cells transformed with the plasmid were grown overnight at 37°C in LB medium containing 50 μg/ml kanamycin. Cell cultures were diluted 1:50 in fresh LB with 50 μg/ml kanamycin and grown to an OD_600_ of 0.6, before adding 0.5 mM IPTG to induce protein expression for 4 h at 37°C. The cells were collected by centrifugation at 4,000 rpm for 20 min at 4°C, resuspended in lysis buffer (10 mM potassium phosphate buffer at pH 7.2, 150 mM KCl, 5 mM imidazole, 5% v/v glycerol, 1 mg/ml lysozyme, a cOmplete™ EDTA-free Protease Inhibitor tablet (Roche), and 1 μg/ml DNase I), and lysed by sonication. The soluble protein was recovered in the supernatant by centrifugation at 20,000 rpm for 50 min at 4°C, and purified by nickel affinity chromatography (Super Ni-NTA agarose resin, Generon) at 4°C, eluting the (His)_6_-SUMO tag with 10 mM potassium phosphate buffer at pH 7.2, 150 mM KCl with 250 mM imidazole. The tag was cleaved by incubating the construct with tobacco etch virus protease (1:5 protein construct/tobacco etch virus molar ratio) overnight at 4°C, while dialyzing the mixture with 10 mM potassium phosphate at pH 7.2, 1 M KCl. A second nickel column at 4°C was applied. The flow-through was collected and dialyzed at 4°C against 10 mM potassium phosphate buffer at pH 7.2 and 15 mM KCl. Pure VHH5 was obtained after a further step of size-exclusion chromatography on an Äkta pure system (HiLoad 16/60 Superdex 75 prep grade column, GE Healthcare). The protein was eluted in 10 mM potassium phosphate buffer at pH 7.2 and 15 mM KCl, aliquoted, and flash-frozen and stored at −20°C. The protein purity was assessed by SDS-PAGE and size-exclusion chromatography.

### Circular Dichroism and NMR Measurements

Far-UV CD spectra of VHH5 (50 μM) was acquired at 25°C in 10 mM potassium phosphate buffer at pH 7.2 and 15 mM KCl. CD spectra were recorded on a JASCO-1100 spectropolarimeter equipped with a temperature control system, and averaged over 10 scans. Measurements were carried out in 1 mm path-length quartz cuvettes (type S3/Q/1; Starna Scientific), applying a constant N_2_ flush at 4.0 l/min. NMR experiments were carried out at 800 MHz on an Avance Bruker spectrometer equipped with a cryogenic probe. The sample (160 μM) was in 10 mM potassium phosphate at pH 7.2 with 15 mM KCl and 10% D_2_O. 1D spectra were acquired at 25°C.

### ELISA Assays

For the Sandwich ELISA, purified VHH5 were coated in triplicates onto a 96-well plate at concentrations of 1 μM, 3 μM, 5 μM, and 10 µM (corresponding to 15–150 μg/ml), left overnight at 4°C, and in carbonate buffer at pH 9.6. After coating, 2 h blocking at room temperature was performed in PBS/BSA at 1% and pH 7.4. Purified RRM1-2, RRM1, and RRM2 (10 μg/ml) were used to capture the VHH5 prey. The solution was incubated for 2 h at room temperature, followed by a further 2 h incubation in the presence of rabbit anti-TDP-43 polyclonal antibodies (Proteintech) at a 1:2000 dilution. Detection of the retained antigen was performed with goat anti-rIgG [HRP] antibody (Cell Signaling) at a 1:2000 dilution. After a 2 h incubation at room temperature in PBS/BSA 1%, with 3,3′,5,5′Tetramethylbenzidine (TMB) (ThermoFisher, cat. No. 34021) the absorbance was read at 450 nm. Antibody dilutions were in PBS/BSA 1%, pH 7.4. The wells were washed three times between steps with PBST at 0.05% and pH 7.4. Wells that did not contain VHH5 but all the other components were used as negative controls.

For the indirect Elisa, purified RRM1-2, RRM1, and RRM2 were coated in triplicates in a 96-well plate at a concentration of 1 µM (corresponding to 10 μg/μl), left overnight at 4°C in carbonate buffer at pH 9.6. After coating, the reaction was blocked for 2 h at room temperature by PBS/BSA at 1%, and pH 7.4. Purified VHH5 (1 μM, 3 μM, 5 µM, and 10 μM, corresponding to 15–150 μg/ml) was used to capture the antigen by a 2 h incubation at room temperature. Detection of VHH5 was performed with rabbit anti-camelid VHH [HRP] antibody (GenScript) at a 1:5,000 dilution. After 2 h incubation at room temperature in PBS/BSA 1%, with 3,3′,5,5′Tetramethylbenzidine (TMB) (ThermoFisher, cat. No.34021) the absorbance at 450 nm was detected. The antibody dilutions were in PBS/BSA 1%, pH 7.4. The wells were washed three times between steps with PBST at 0.05% and pH 7.4. Wells that did not contain the antigen (TDP-43 fragments) but all the other components were used as negative controls.

## Results

### Naïve Llama VHH SPLINT Library Construction

**FIGURE 1:**
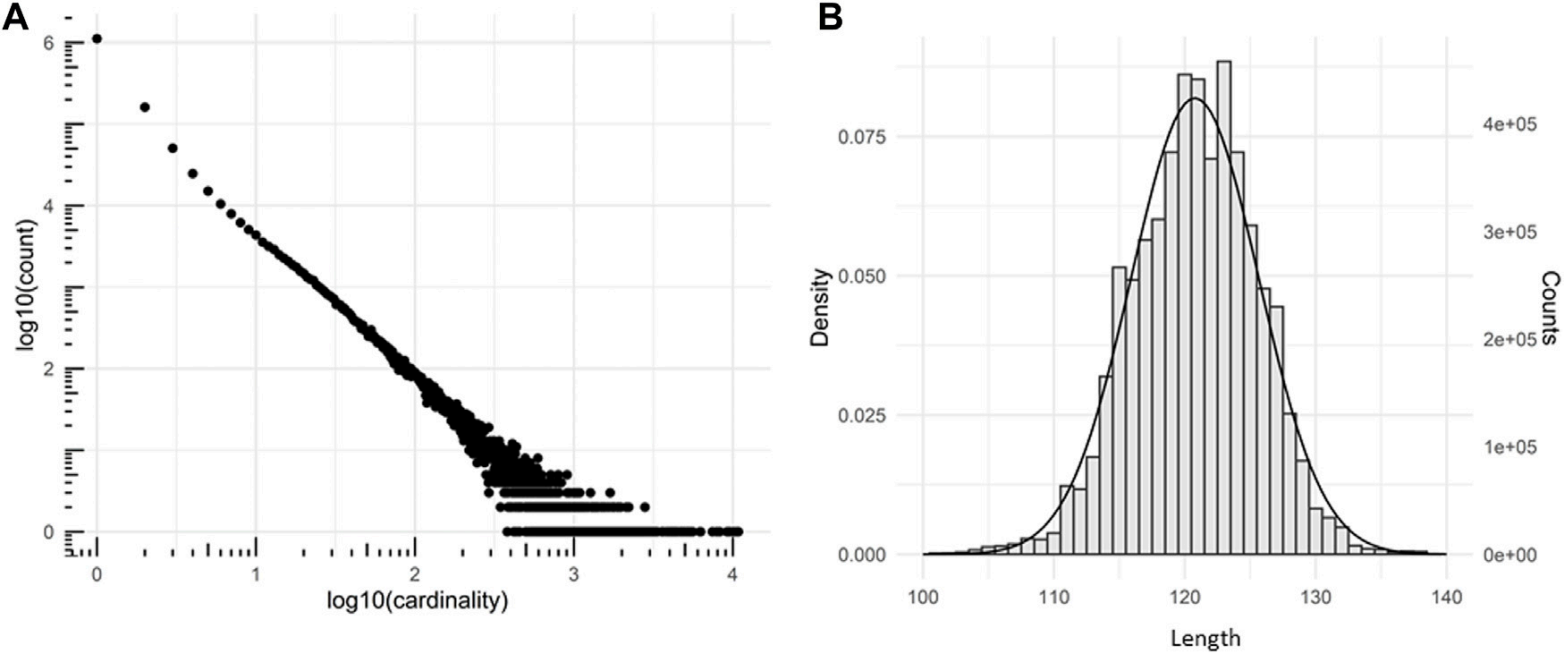
Characterization of the SPLINT library. **(A)** Cardinality plot of the sequenced library. Log-log plot showing the number of time a group of identical n sequences (*n* = cardinality) was found in the sequencing. **(B)** VHH proteins length distribution. Distribution of the number of residues observed in the peptide chains of the of translated llama VHHs (amino acid sequence length) and gaussian fit.

A VHH library was created from cDNA derived from peripheral blood lymphocyte RNA isolated from two not immunized (naïve) *llama glaba* animals and cloned in SPLINT format (Visintin et al., 2004) for further use. In this format, the VHH antibody domains are fused in frame to the activation domain of the transcription factor VP16. The VHH DNA library was amplified in bacteria obtaining a complexity of 1.7 × 10^7^, defined as the number of total transformants, determined through colony forming unit (CFU) count. The library was sequenced by Next Generation Sequencing. From a total number of 6,322,129 sequences the sequence diversity resulted to be 1.15 × 10^6^. The library complexity was estimated by the truncated Negative Binomial distribution (Fantini et al., 2017) to fit the number of sequences as a function of sequence cardinality (Figure 1A). Most of the sequences (93%) were full-length and did not contain premature stop codons or frameshifts. The VHH lengths fit a normal Gaussian distribution centered on 120.7725 amino acids with a standard deviation of 4.8723 (Figure 1B). The diversity of the SPLINT library is in line with our previous mouse or human libraries, which were shown to contain antibody domains able to effectively bind their corresponding protein antigen intracellularly.

### Intrabody Selection

**FIGURE 2:**
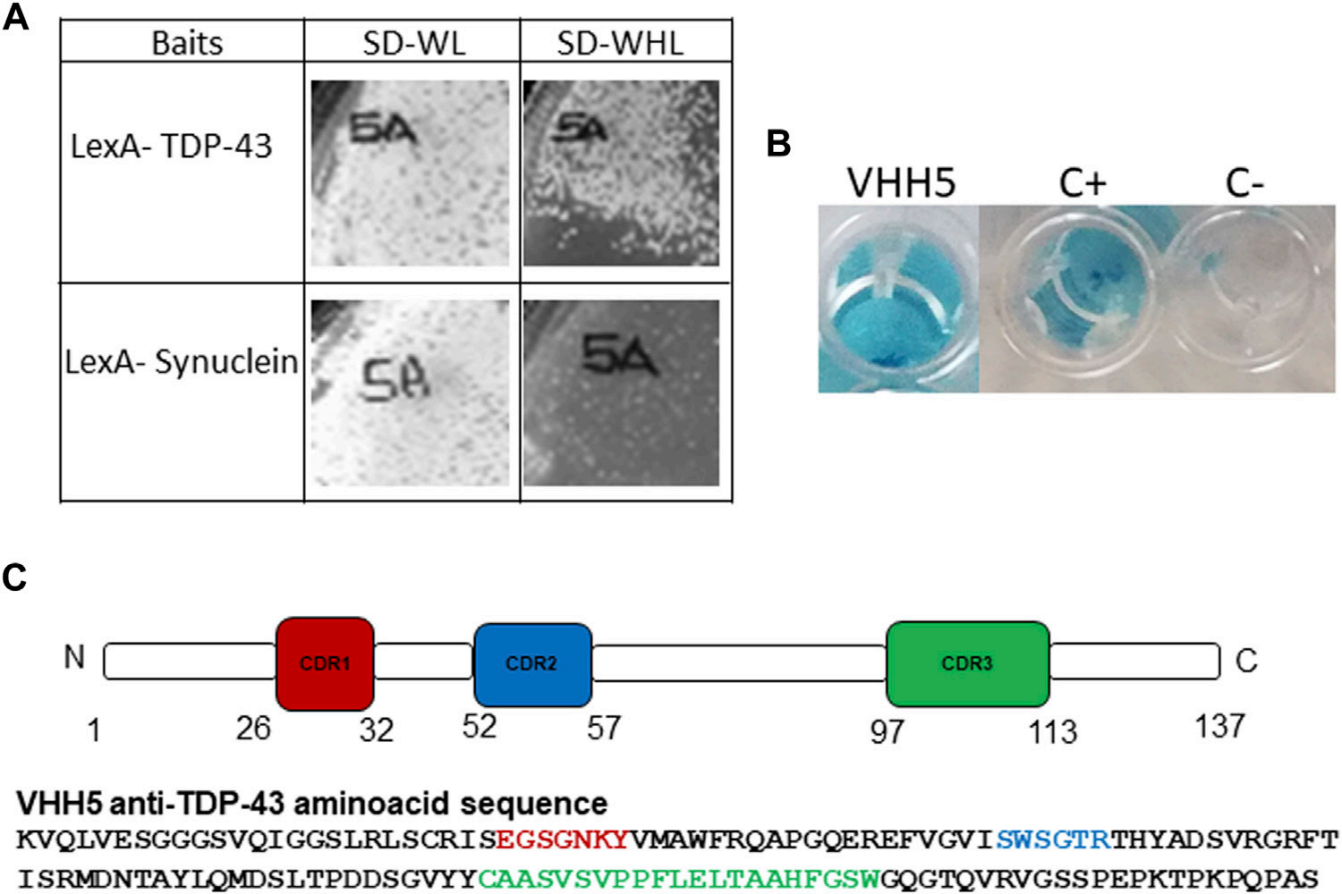
Selection of VHH5. **(A)** Growth on selective plate SD-WHL of the VHH5 co-transformed with the LexA-TDP-43 bait and the unrelated LexA-Synuclein bait. The images of growth on plates were acquired using Chemidoc XRS (Biorad). **(B)** Liquid β-gal assay of yeast co-expressing the LexA-TDP-43 bait and the VHH5 intrabody, C+: LexA-TDP-43+ Y1, an anti-LexA intrabody, and C−: LexA-TDP-43+ scFv anti-pTau. The images were acquired using HUAWEI Mate 10 lite. **(C)** Amino acid sequence of VHH5 and schematic representation of VHH5 with the position of the CDRs, as defined using the Chothia and Lesk numbering scheme (Chothia et al., 1989) in the http://cao.labshare.cn/AbRSA/abrsa.php server (Li et al., 2019).

The yeast two hybrid based IACT system was used to select intracellular specific intrabodies against TDP-43 from the VHH SPLINT library (Visintin et al., 2002; Visintin et al., 1999). IACT screening works by exploiting yeast L40 strains co-transformed with antigen-bait/antibody-prey pairs, in which the antigen-bait is fused to a DNA binding domain (LexA-DBD) that is challenged with a library of natural recombinant antibody domains fused to the VP16 activation domain (the prey). The TDP-43 gene (amino acids 1–414) was cloned in fusion with LexA and used to challenge the llama antibody library (Visintin et al., 2004). A positive interaction between a prey and the bait activates transcription of the *HIS3* gene, allowing survival on selective media (SD-WHL), and of the *LacZ* gene as a second marker of interaction. After a primary selection, a second round of selection pointed to a lead candidate (VHH5) as a positive TDP-43 interactor. The specificity of VHH5 was analysed for survival on selective media (SD-WHL) using either the screening bait (LexA-TDP-43) or an unrelated bait (LexA-Synuclein) to exclude interactions between VHH5 and the LexA domain of the fusion protein bait (Figure 2A). Activation of the second reporter marker *LacZ* was assessed in a liquid β-gal assay*.* VHH5 interaction with LexA-TDP-43 gave positive β-gal assay as compared to the positive control of the assay (interaction of LexA-TDP-43 with the Y1 anti Lex A nanobody) and the negative control (interaction of LexA-TDP-43 with a scFv anti p-Tau) (Figure 2B). Analysis of the intrabody sequence revealed a short charged H1 loop, a shorter H2 loop containing a Trp, and a rather long H3 loop, comprising 17 residues according to Chothia, and Lesk numbering system (Chothia et al., 1989). This loop is circa ten residues longer than the average of the H3 in antibodies, but within average for intrabodies (Figure 2C). It does however contain many degrees of freedom, making prediction of its structure not straightforward.

### Attempts to Characterise Recombinant VHH5 by *E. Coli* Overexpression

In the attempt to characterize the anti-TDP-43 VHH5, we tried to express and purify the protein in *E. coli*. VHH5 was first inserted into a pET-17b expression vector fused with the PelB leader sequence that directs proteins to the periplasmic space allowing disulfide bridge formation. The construct was transformed in *E. Coli* BL21 (DE3) cells but resulted poorly overexpressed (Supplementary Figure S1A). We then re-cloned the protein in a pET-17b plasmid as fusion protein with an N-terminal SUMO solubilization domain and a (His)_6_-tag to enhance protein solubility. We also changed the *E. coli* strain and expressed it in BL21 (DE3) pLysS cells. The expression yield appreciably increased but the highly expressed protein accumulated in the cytoplasm as inclusion bodies (data not shown). All attempts to avoid precipitation failed, including changes of the induction temperature. Inclusion body formation has been proven to result from the conflict between aggregation and protein fold and it is a well-known impediment particularly in antibody production (Ventura and Villaverde, 2006).

To predict which residues/regions of the protein could contribute to aggregation, we analysed the sequence by AGGRESCAN (Conchillo-Solé et al., 2007). This is a web-based software that allows prediction of the aggregation properties of a protein on the basis of its sequence. We found several regions predicted to be aggregation prone, some of which in the CDR loops (Supplementary Figure S1B). As an alternative strategy, we resorted to model the structure of the intrabody by comparative modelling to have an independent insight based on a 3D model of the structure of VHH5 and a more solid idea of the expected structural features.

### Modelling the Antibody Scaffold

The structure of the antibody main scaffold, that is the β-sandwich that holds the antigen recognizing CDR loops, can be easily predicted as this region is highly conserved amongst antibodies, and their derivatives (Narciso et al., 2011). A BLAST search over the PDB database identified 5wcc as the closest sequence-wise template for comparative modelling. This is the crystal structure of the broadly neutralizing Influenza A antibody VRC 315 02-1F07 Fab. We used in parallel both the SWISS-MODEL (Waterhouse et al., 2018) and the ABodyBuilder (Leem et al., 2016) servers for the prediction. SWISS-MODEL relies on ProMod3, an in-house comparative modelling engine based on OpenStructure (Biasini et al., 2013). The ABodyBuilder algorithm also follows template selection, orientation prediction, and CDR loop modelling and side chain prediction. ABodyBuilder then annotates the “confidence” of the model as the probability that a component of the antibody (e.g., a loop or a strand) is modelled within a RMSD threshold. We obtained models that were closely evaluated. The two energetically best structures from each of the two programs could be superposed with a RMSD of 0.45 Å (Supplementary Figure S2). The template and target structures were of similar lengths with two one-residue insertions in the H2 and H3 CDR loops and a deletion in another loop.

The two energetically best structures from each of the two programs were then refined by energy minimization using the CHARMM36m force field that has extensively been shown to be robust in simulations of globular proteins. Twenty thousand steps of conjugated gradient energy minimization were applied using no constraints or with positional constraints on the backbone heavy atoms and on the heavy atoms of the solute in the regions 1–70 and 77–135 for both the SWISS-MODEL and ABodyBuilder VHH5 structures 1, 2, and 3. The resulting models were then used as the input to model the CDR loops of VHH5.

### H3 Modelling and Structure Refinement

The challenge in antibody structure prediction is the design of the CDR loops. Of the three loops, H1 and H2 can easily be classified according to the canonical structures first described in 1987 by Chothia and Lesk, and their structures can confidently be predicted (Al-Lazikani et al., 1997). The problematic loop is H3 because of the high variability of its sequence, length, and conformation that makes difficult to build a high-quality structure with ordinary modelling techniques. Modelling of the H3 loop (residues 94–114) was carried out using the Sphinx algorithm, a combination of the FREAD knowledge-based method (Deane and Blundell, 2001; Choi and Deane, 2010) and an *ab initio* algorithm*.* Given the overall similarity between the two structural bundle and to reduce the number of structures to analyse, we restricted the prediction only to the best structure from SWISS-MODEL. We obtained a bundle of 500 structures from which we selected 10 energetically best structures. In most of the solutions the loop turned out not to contain any regular structural element with the loop mostly protruding out from the rest of the molecule (Supplementary Figure S3). Only in one model, the loop contains a short 1-turn helical element in the middle of the loop. In seven out of ten structures, and the first two residues of the loop pair with a close-by strand.

**FIGURE 3:**
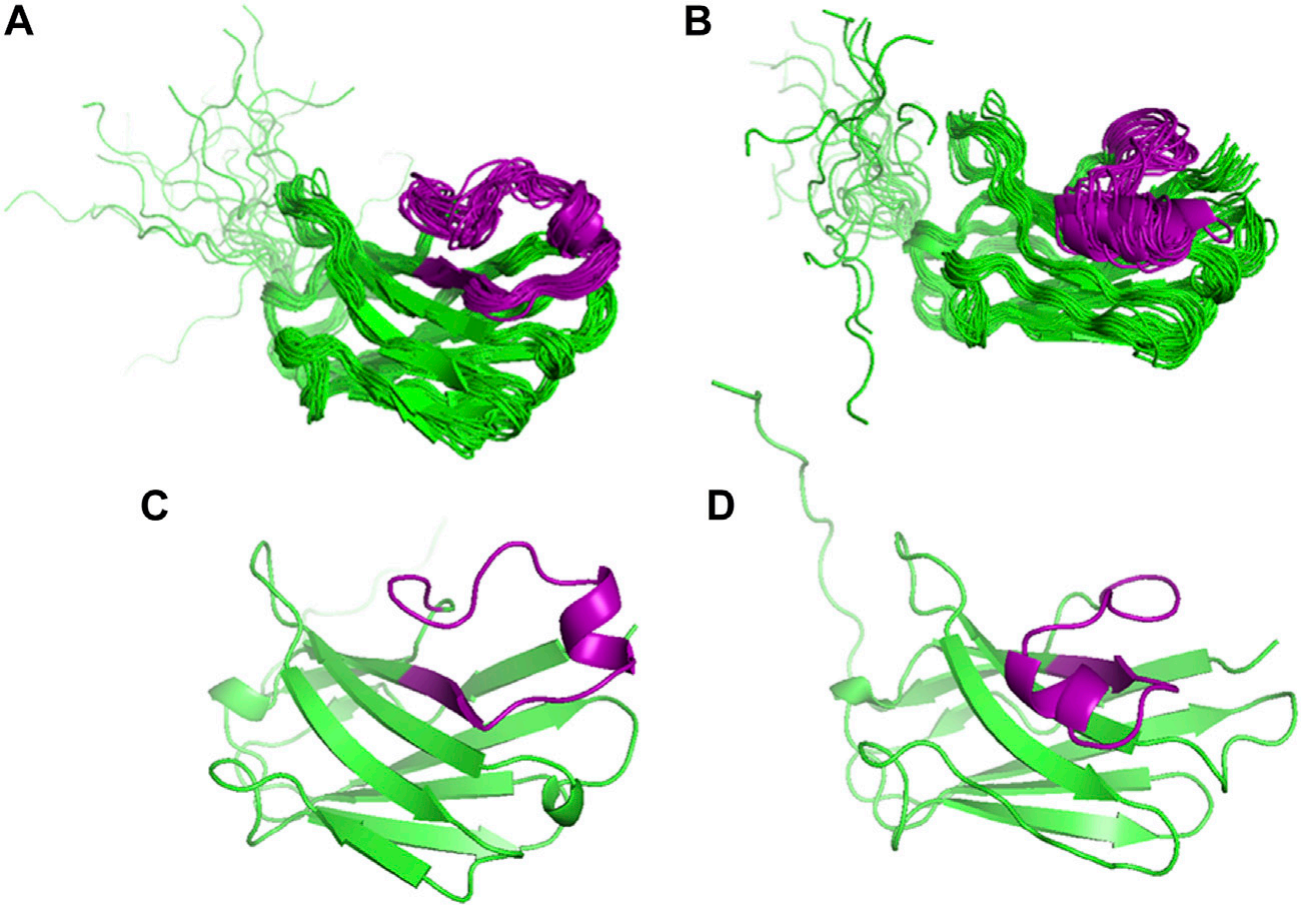
Comparison of the MD derived ensembles of VHH5 Model 1 from the H3 loop generation. **(A)** Twenty structures from 1.4 to 39.8 ns; **(B)** Nineteen structures from 43.2 to 80.0 ns of the simulation time. The H3 loop conformations obtained from the MD simulations; **(C)** open conformation; **(D)** closed conformation. The H3 loop is colored in purple.

**FIGURE 4:**
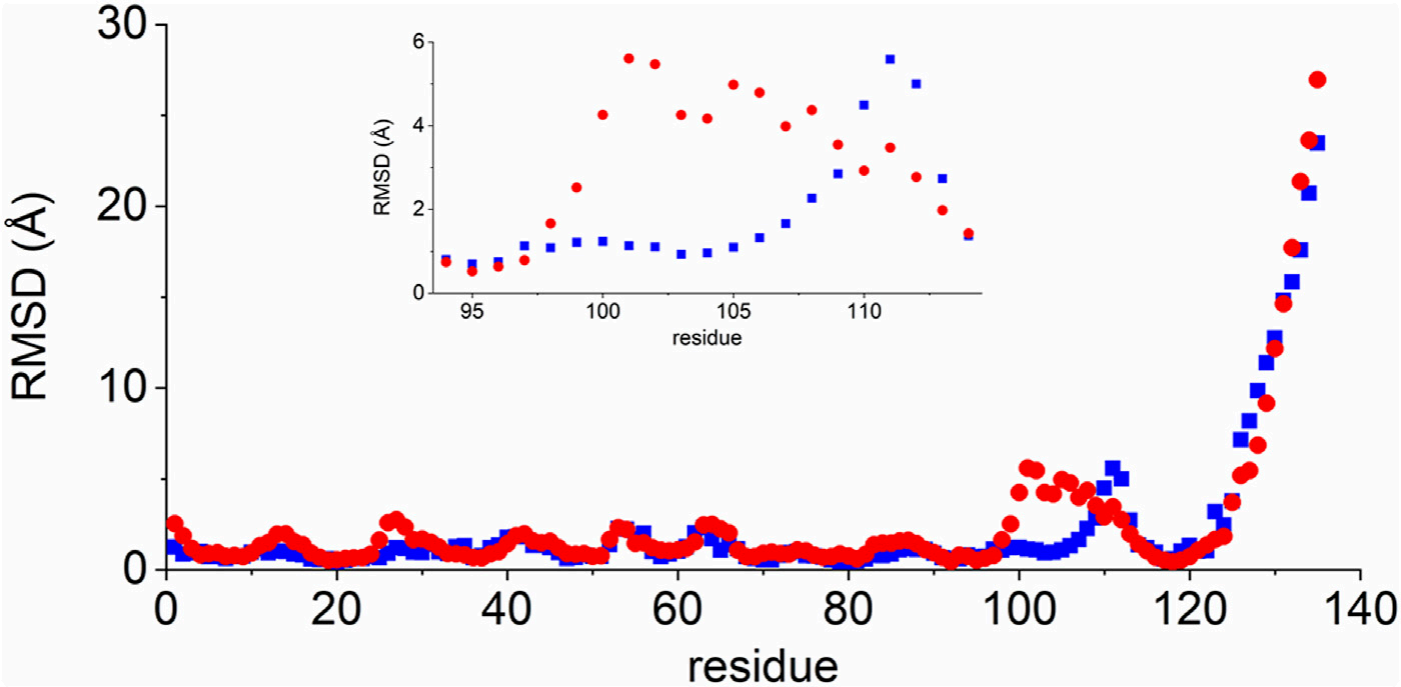
RMSD values of VHH5 Model 1 after the H3 loop generation for the two loop conformations. The RMSD was calculated for the CA, C′, and N backbone atoms of each residue. *Blue rectangles*: open conformation, 1.4–39.8 ns; *Red dots*: closed conformation, 43.2–80.0 ns. RMSD values for the residues in the H3 loop region are shown in the insert.

We then refined the energetically most favourable structure from the H3 loop modelling (Model 1) by MD simulations, also to obtain information on the conformational space covered by the long H3 loop. Throughout the 80 ns production run, this loop adopted two significantly different conformations: protruding out from the rest of the molecule (open form, 1.4–39.8 ns) or bending closer to the beta strands encompassing residues 33–38 and 46–52 (closed form, 43.2–80.0 ns) (Figure 3). This potential variability was also reflected in the time evolution of the total RMSD calculated for the N, CA, and C backbone atoms (Supplementary Figure S4). When the RMSD of the individual residues was separately calculated along the trajectory for each of the open and closed forms (Figure 4), variability was noticed at the three CDR loops, and especially at H3. The C-terminus (residues 122–135) is completely disordered.

The predicted models were validated by PROCHECK (PDBSum) (Laskowski et al., 1996; Laskowski, 2001). According to this analyser, the Ramachandran plot contained 90% of the residues in the most favoured regions, and 10% in additional/generously allowed regions (Supplementary Figure S6). Gly and Pro residues were also located in allowed regions. The G-factors on dihedral angles, that provide a measure of how unusual, or out-of-the-ordinary, a property is, were all above the −0.5 threshold or positive, and indicating good quality. The overall average value was −0.14.

### Structure-Guided Optimization of VHH5 Expression

**FIGURE 5:**
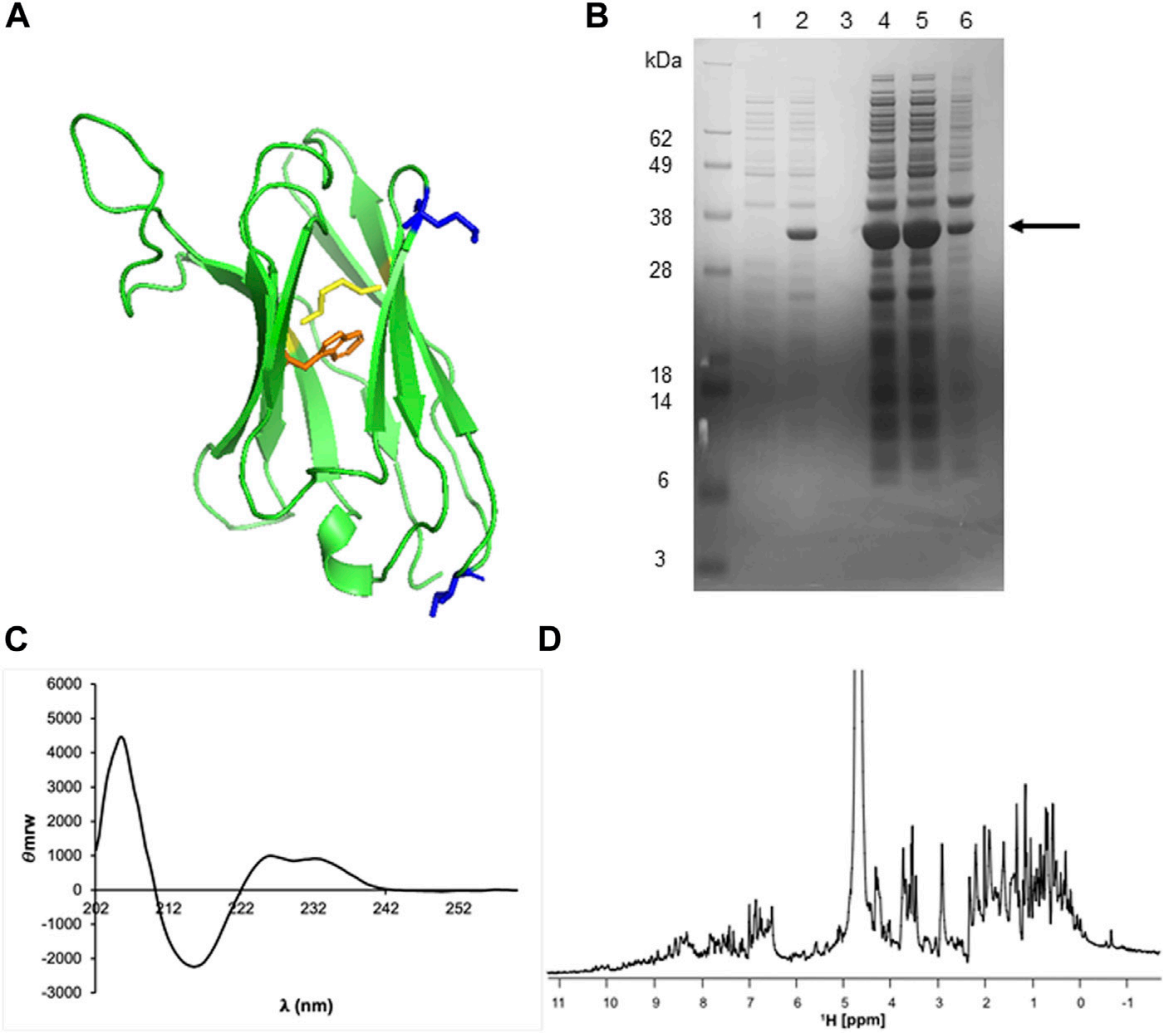
Structural analysis, production, and characterization of VHH5. **(A)** VHH5 structure. The disulfide bond is highlighted in yellow and the side chain of the tryptophan is in orange. The two hydrophobic residues, I15 and M74, that were hypothesized to help inclusion body formation are highlighted in blue. **(B)** Overexpression of VHH5 in *E. Coli* BL21 (DE3)pLysS cells as a soluble protein. SDS-PAGE analysis of SUMO + VHH5 (29 kDa) shows the soluble protein and a high overexpression. The columns correspond to: lane 1, pre-induction; lane 2, after induction with IPTG; lane 3, pre-lysis supernatant; lane 4, pre-lysis pellet; lane 5, post-lysis supernatant; lane 6, post-lysis pellet. **(C)** CD and **(D)** ^1^H NMR spectra of VHH5 recorded at room temperature.

We used the predicted structures to analyse the protein surface and identify exposed hydrophobic residues not contributing to the hydrophobic cores or to the CDR loops that could be mutated to reduce the risk of the proteins to be in inclusion bodies. We both visually inspected the models and analysed the coordinates with the DSSP software which provides per residue accessible surface areas. As the result of this analysis, we found that the regions that could mostly promote aggregation could be H3 which is indeed rather hydrophobic with four bulky hydrophobic residues and two uncharged aromatics. This region cannot however be mutated as it may be essential for epitope recognition. Additionally, we found a few exposed hydrophobic residues such as I15 and M74 that could potentially interfere with protein folding leading to inclusion bodies (Figure 5A). We thus decided to mutate I15 to alanine and M75 to lysine creating the double mutant VHH5-I15A_M75K and attempted to express this mutant in *E. coli*.

We found that protein production switched from being all in the inclusion bodies to being mostly soluble (Figure 5B). This strategy allowed us to obtain suitable quantities of VHH5-I15A_M75K. After purification, we managed to typically obtain ca. 13 ml (1.96 mg/ml or 132 µM) of >98% pure protein after cleaving it from the tag. The protein identity was confirmed by mass spectrometry which also confirmed disulfide formation (data not shown). We also confirmed the state of fold by far-UV circular dichroism (CD), a technique able to detect the secondary structure of proteins. The CD spectrum of VHH5- I15A_M75K recorded at room temperature has a maximum at 205 nm and a single minimum around 215 nm which are features typical of the β-sheet conformation expected for an antibody (Figure 5C). The positive contribution at 225–235 nm is usually diagnostic of the presence of stacking interactions between aromatic residues (Budyak et al., 2013). The mono-dimensional NMR spectrum of the unlabelled protein presented well dispersed resonances as expected for a folded monomeric protein of the size of VHH5 (Figure 5D). We thus concluded that the protein obtained was folded and well-behaved.

### Epitope Mapping

**FIGURE 6:**
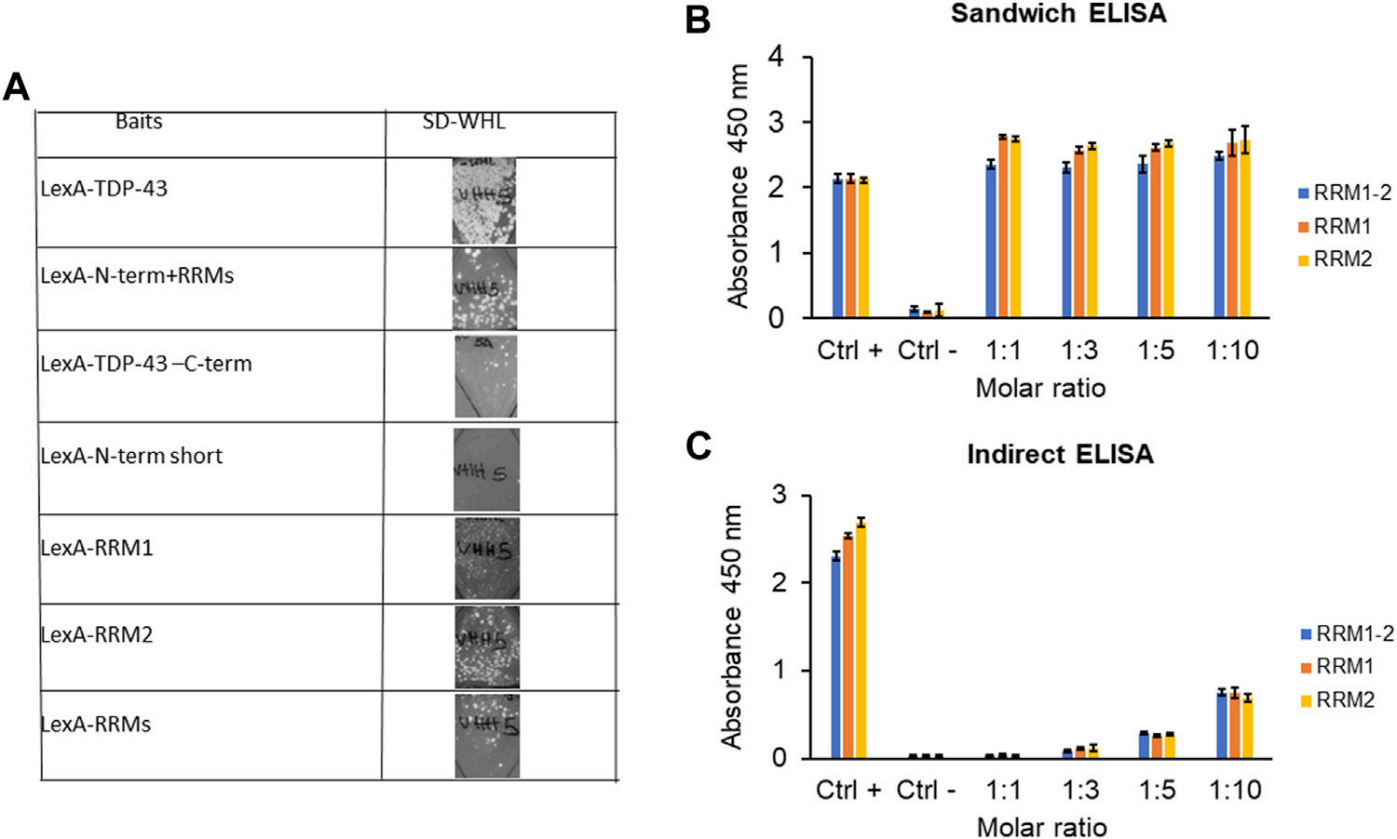
Epitope mapping of VHH5 on TDP-43. **(A)** *In vivo* Epitope Mapping. The VHH5 was transfected in L40 yeast strain expressing the baits LexA-TDP-43 full length (residues 1–414), LexA- N-Term + RRM1-2 (1–258), N-term short (1–105), RRM1 (106–176), RRM2 (192–258), and RRM1-2 (160–208). Interaction is detected by growth on–WHL plates. **(B)** Sandwich ELISA assay. Coating antibody: VHH5 (final molar ratio coating antibody: binding antigen 1:1, 1:3, 1:5, and 1:10); Binding antigen: RRMs, RRM1, and RRM2 (1 µM); Detection: anti-TARDBP and then anti-hIgG-HRP. The assay shows an interaction of VHH5 with all the TDP-43 fragments. **(C)** Indirect ELISA assay. Coating antigen: RRM1-2, RRM1, and RRM2 (1 µM); Binding antibody: VHH5 (molar ratio 1:1, 1:3, 1:5, and 1:10); Detection: anti-VHH-HRP. The assay shows an interaction of VHH5 with all the TDP-43 fragments. The interaction increases as the molar ratio increases.

To characterize the epitope of TDP-43 recognized by VHH5, we first performed *In Vivo* Epitope Mapping (IVEM) in yeast (Visintin et al., 2002) by truncating the original LexA-TDP-43 bait into two fragments, LexA-N-term + RRM1-2 (residues 1–258) and LexA-C-term (residues 259–414). The epitope recognized by the VHH5 resulted to be located in the N-terminal half of the protein. To further narrow the region carrying the epitope, a second IVEM was carried out by splitting this region into four smaller baits containing the N-terminus (1–105), RRM1 (106–176), RRM2 (192–258), and a fragment of RRMs (160–208). The epitope seemed to be mainly located in RRM2, since growth on SD-WHL plates was detected both with the LexA-RRM2 and the RRMs baits (Figure 6A). To substantiate these results with further evidence, we used the purified recombinant VHH5- I15A_M75K for ELISA experiments. We performed both sandwich and indirect ELISA assay using a rabbit anti-TDP-43 polyclonal antibody (Proteintech) and a rabbit anti-camelid VHH [HRP] antibody (GenScript) respectively. In both cases, we observed response to RRM1, RRM2, and RRM1-2, indicating that the epitope involves both domains (Figures 6B,C). This result could mean that VHH5 recognises each of the repeats which share some homology. However, while the homology is fairly high, and the sequence identity is only 26%. It is thus fairly unlikely that there are two independent epitopes one in each repeat. It is more likely that the epitope is conformational and involves both domains. We also noticed that only the indirect ELISA showed a dependence on the antibody to protein ratios. This could be explained by considering that the difference between the two assays is that in the indirect ELISA, the target protein is fixed and the intrabody is added at increasing concentrations. No concentration dependence in the latter assay could easily be explained by the assumption that when the intrabody is fixed it could adopt a conformation that makes it more competent for binding. Viceversa, when the target protein is fixed, the epitope may be partially masked. This means that the detected affinity can be different in the two cases. Thus, the signal can appear saturated in Figure 6B but not in the indirect ELISA done with the intrabody in solution.

**FIGURE 7:**
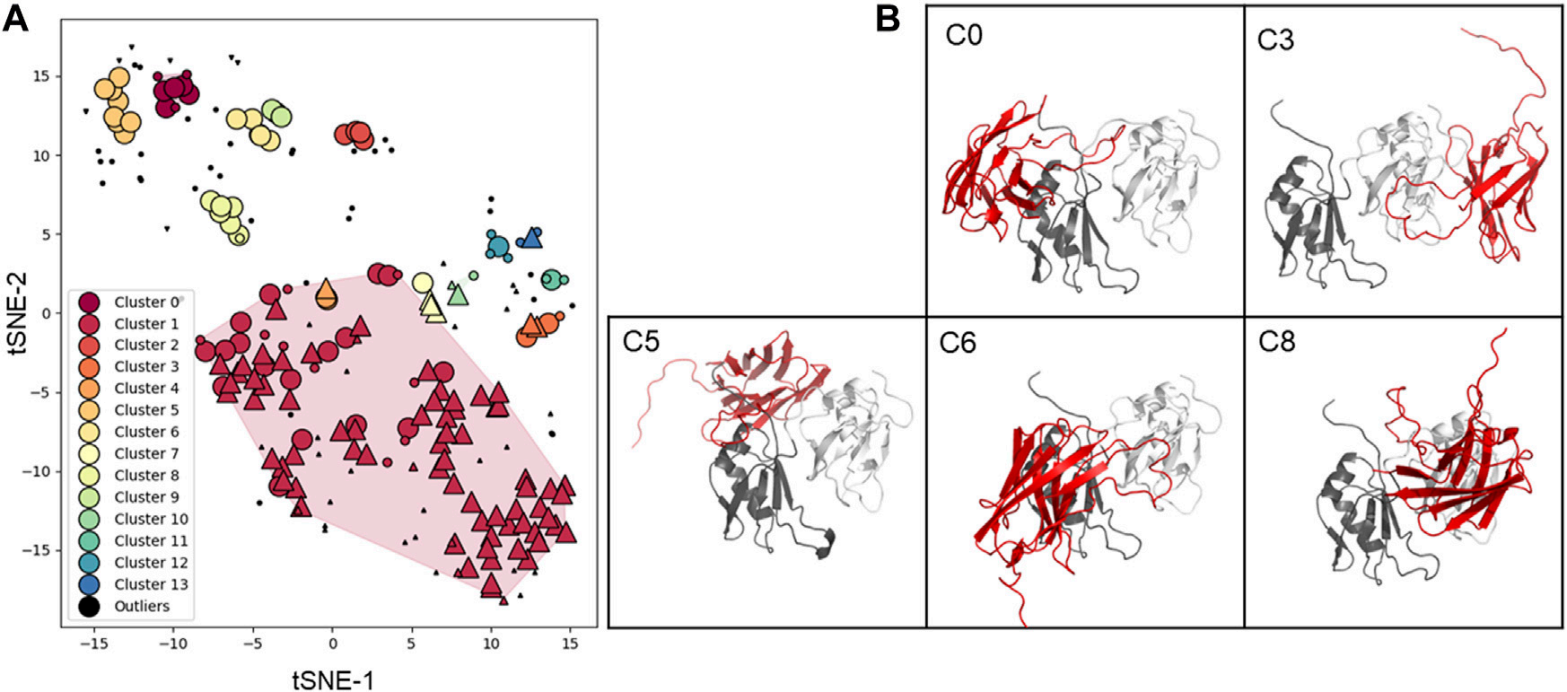
Clustering and structure of the docking solutions. **(A)** Clustering is represented as a 2D map that preserves local similarity. Each dot corresponds to a docking solution, and is coloured according to the cluster it belongs to. Dots depicted as upward triangles, downward triangles, and circles represent solutions where the antibody interacts with the first (RRM1), second (RRM2), and or both antigen domains (RRM1-2), respectively. Solutions depicted in black are considered outliers by the clustering algorithm, small dots, and large dots are core and reachable elements, respectively. **(B)** Representative solutions from clusters with more than five elements, excluding cluster 1. The antibody is represented in red, and the first and second antigen domains in dark grey and white, respectively.

Using this information, we then performed molecular docking. Although docking carried out on low resolution structures and without experimental restraints has only very limited reliability, we reasoned that it could provide a visual impression of epitope binding and inform future studies. Models of the antigen-antibody complexes were generated by the ClusPro software using each of the ten energetically best Sphinx structures and the NMR structure of the putative antigen (PDB 4bs2). This calculation resulted in 228 models which were further analysed. After the filtering procedure described in the Materials and Methods section, a total of 14 clusters were identified (Figure 7A). The complex structures with the lowest score and binding free energy were selected and analysed (Figure 7B). Cluster 1 contains the vast majority of the solutions, in which the antibody only interacts with a single domain of the antigen. However, upon closer inspections, we realised that these solutions were likely the result of an artefact of the docking procedure: the H3 loop of the antibody would encircle the C-terminus of the antigen, in a configuration that would result in a knot or a lasso in the complete antigen. Excluding these solutions, cluster 0, 2, 5, 6, 8, 9, 11, 12, and 13 mainly contained solutions in which the interaction involved both domains. In total, 51 out of the 61 solutions that were not outliers nor part of cluster 1, and contained interactions to both domains (Figure 7B). These models, that are only indicative and low resolution, will need experimental validation through fine epitope-mapping at the level of the individual residues.

## Discussion

The use of antibodies in misfolding diseases is in principle a flexible and ductile strategy to control protein aggregation, because, by binding to a monomeric protein, they prevent self-assembly by steric hindrance. There are now several different strategies that allow screening (Hanes and Pluckthun, 1997; Smith and Petrenko, 1997; Ho and Pastan, 2009; Uchanski et al., 2019), ab initio design (Hardin et al., 2002; Zhu and Day, 2013) or evolutionary selection of antibodies, and smaller derivatives (Visintin et al., 2002). A problem remains however the production of the antibody by bacterial expression once a potentially effective sequence has been identified. Unfortunately, the large molecular weight (typically ∼150,000) and hetero-tetrameric composition of antibodies with two different polypeptides (a heavy and a light chain) and a total of up to 15 disulfide bridges make difficult when not prohibitive their production in bacteria or in the cytoplasm of eukaryotic cells. This is why scFv fragments, that contain only one copy of the variable domains of immunoglobulin motif, offer undiscussable advantages. However, also in this case, it is difficult to predict *a priori* whether an intrabody obtained by library screening can easily be produced in *E. coli*, and problems in successfully refolding the intrabody from inclusion bodies have been described (Vaks and Benhar, 2014; Bao et al., 2016).

In the present study, we used a composite approach in which we screened an intrabody for TDP-43 recognition, and produced it in bacteria and characterised it for epitope recognition. We first described a new naïve library of llama VHHs, and exploited it to select a new anti-TDP-43 VHH directly from the TDP-43 cDNA. A significant advantage of SPLINT-derived antibodies, as the anti-TDP-43 VHH5 described here, is that the genes coding for the antibody domains are by definition well validated as intrabodies, since the IACT selection is performed under conditions of intracellular expression in yeast cells. SPLINT-derived antibody domains are well suited to be used as intrabodies (Biocca et al., 1990), possibly coupled to effector domains for targeted degradation (Melchionna and Cattaneo, 2007; Schapira et al., 2019) or for imaging purposes.

We then modelled the structure of the intrabody to get a visual impression of its structure. The model suggested exposed hydrophobic residues that could be mutated to reduce the risk of inclusion body formation. We found that it was sufficient to mutate two exposed hydrophobic residues to have a soluble protein that could be purified in suitable amounts for proper direct characterization. We demonstrated by CD and NMR studies that the protein is folded and monomeric and that has all the features expected for the expected β-rich structure. We then demonstrated by ELISA experiments that the double mutant is still able to recognise the TDP-43 epitope. This conclusion was far from being obvious, since it is known that regions outside the CDR loops can contribute to epitope recognition of intrabodies (Sela-Culang et al., 2013). We mapped the epitope binding regions first coarsely by *in vivo* epitope mapping and then, more specifically, and by ELISA experiments with individual or tandem domains of TDP-43. We found that the anti-TDP-43 VHH5 intrabody binds both RRM1 and RRM2. This is in agreement with structural studies that have revealed that VHHs often tend to recognize concave surfaces of their antigens with high shape-complementarity. Based on these experimental findings, we modelled the interaction by *in silico* docking. Despite their overall diversity, in most of the solution we found the long H3 of VHH5 protruding out from the body of the antibody and docks into the cleft formed by the interface between the two domains. This arrangement would permit recognition of the antigen with high shape complementarity. A similar type of recognition has been described in a structural study that compared the binding mode of VHH with that of Fvs using hen egg lysozyme (HEL) as a model antigen (Akiba et al., 2019). Several more studies have also revealed that VHHs usually target concave surfaces on the antigen molecule (Kromann-Hansen et al., 2016; Rossey et al., 2017; Gulati et al., 2018). It is believed that in this way, VHHs compensate for the limitations of their small size, while maintaining the high affinity and specificity that constitute the hallmarks of antibodies.

It is interesting to compare our intrabody with previously developed anti-TDP-43 antibodies. A systematic survey in 2015 revealed the existence of 29 antibodies, many of which were generated in house (Goossens et al., 2015). Amongst the ten highest-ranking primary antibodies, one has two distinct epitopes, that recognize TDP-43 N-terminus and RRM2. Two other antibodies are directed at RRM2, and three have epitopes in the C-terminus of TDP-43. The remaining four antibodies also map in the C-terminus but are specific for phosphorylated serine residues. The majority of these antibodies are polyclonal and therefore their genes cannot be available for further downstream engineering. A single chain antibody against RRM1 was generated in 2019 (Pozzi et al., 2019). Two more monoclonal antibodies were recently described that were raised against an epitope within the RRM2 domain of TDP-43 (residues 198–216) (Trejo-Lopez et al., 2020).

The novel intrabody will aid in diagnostic and research efforts within the context of TDP-43 proteinopathies. Availability of this intrabody opens new avenues to the diagnosis and treatment of ALS.

## Data Availability

The original contributions presented in the study are included in the article/Supplementary Material. Further inquiries can be directed to the corresponding authors.
