# Supplementary material for: Selection and Modelling of a New Single-Domain Intrabody Against TDP-43
Source: Front Mol Biosci. 2022 Feb 14;8:773234. doi: 10.3389/fmolb.2021.773234 (PMC8884700; doi:10.3389/fmolb.2021.773234)
Supplement: Supplementary file 1 [file DataSheet1.docx]

**Supplementary Materials**

**Figure S1 –** Attempts to produce recombinant VHH5 in *E. coli*. A) SDS-PAGE analysis of PelB+VHH5 (18kDa) showing poor expression of the construct. B) Plot of the aggregation-prone regions along the VHH5 sequence as predicted by AGGRESCAN. Peaks above the hotspot threshold line are supposed to promote aggregation.

**Figure S2** – Modelling of the intrabody scaffold. The two best models according to SwissModel (left) and ABodyBuilder (middle) and their superposition (right). The two models were superposed on the backbone atoms of the scaffold.

**Figure S3** – Modelling of the H3 loop. The energetically best ten structures obtained by the Sphinx program are displayed. The structures were first superposed on the backbone atoms of the scaffold residues and then translated. The H3 loop is shown in magenta. Arrows and ribbons indicate beta strands and helical elements.

**Figure S4** – RMSD values calculated for the CA, C’ and N backbone atoms of residues 1-121 of VHH5 Model 1 along the dynamics after H3 loop generation.

**Figure S5** – Ramachandran plot of resulting model after modeling of H3 with Sphinx.

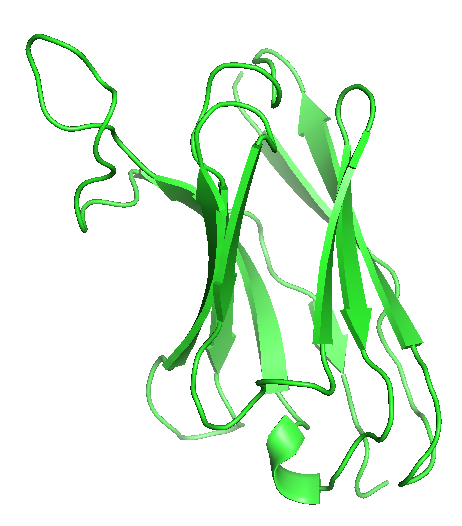

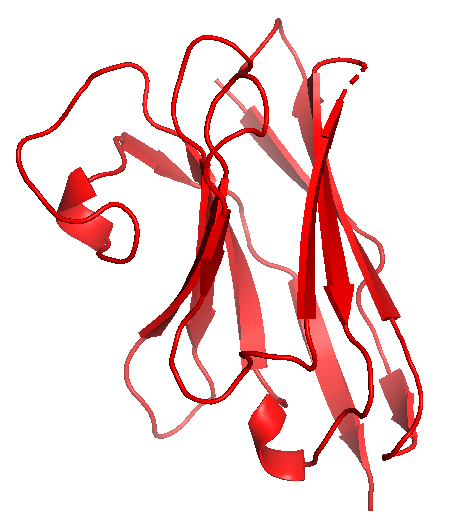

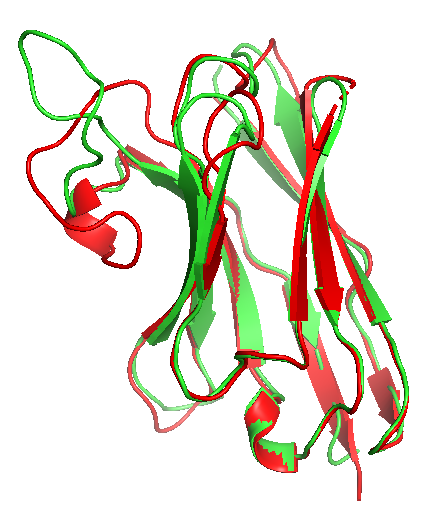


Figure S2


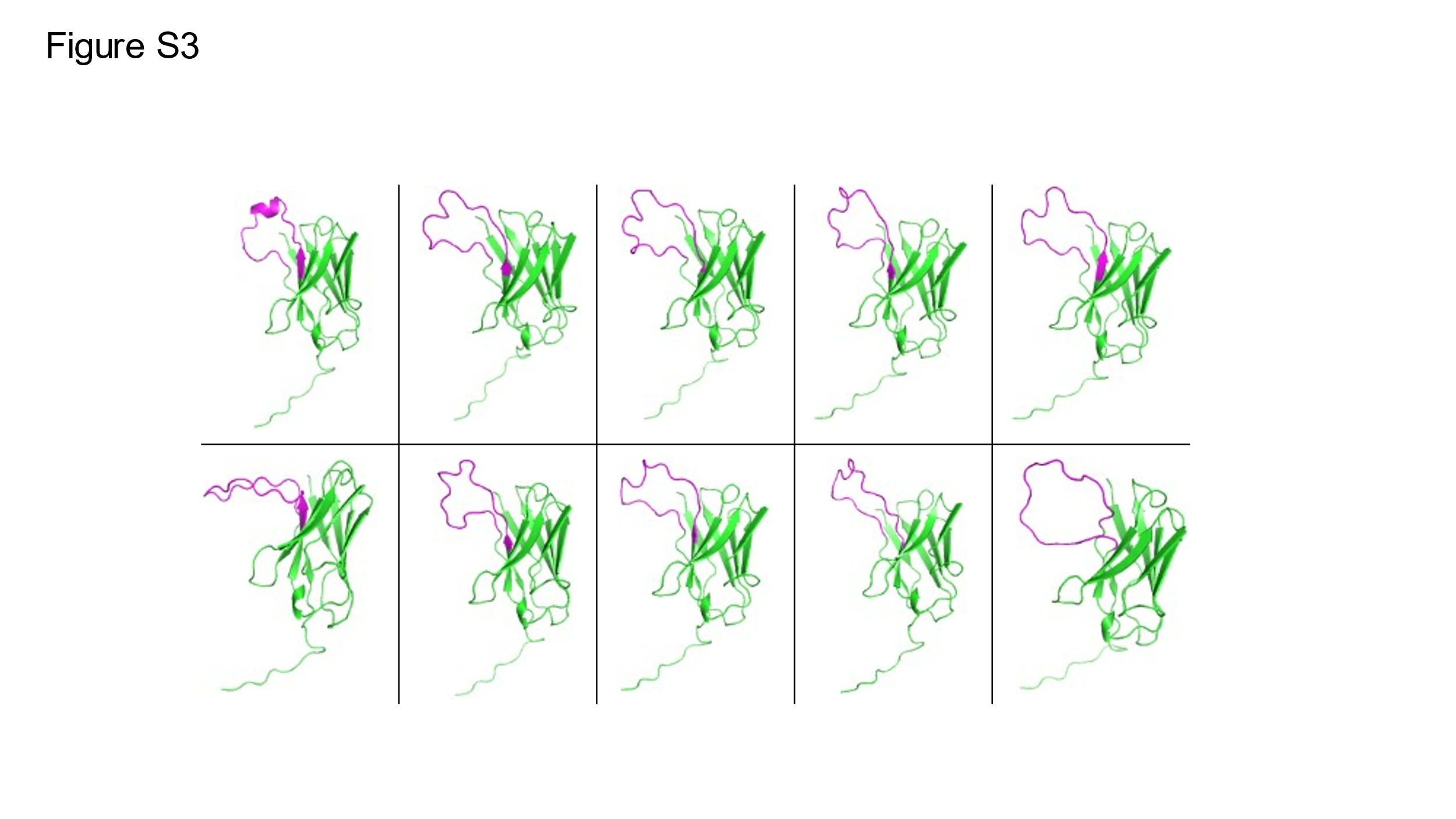


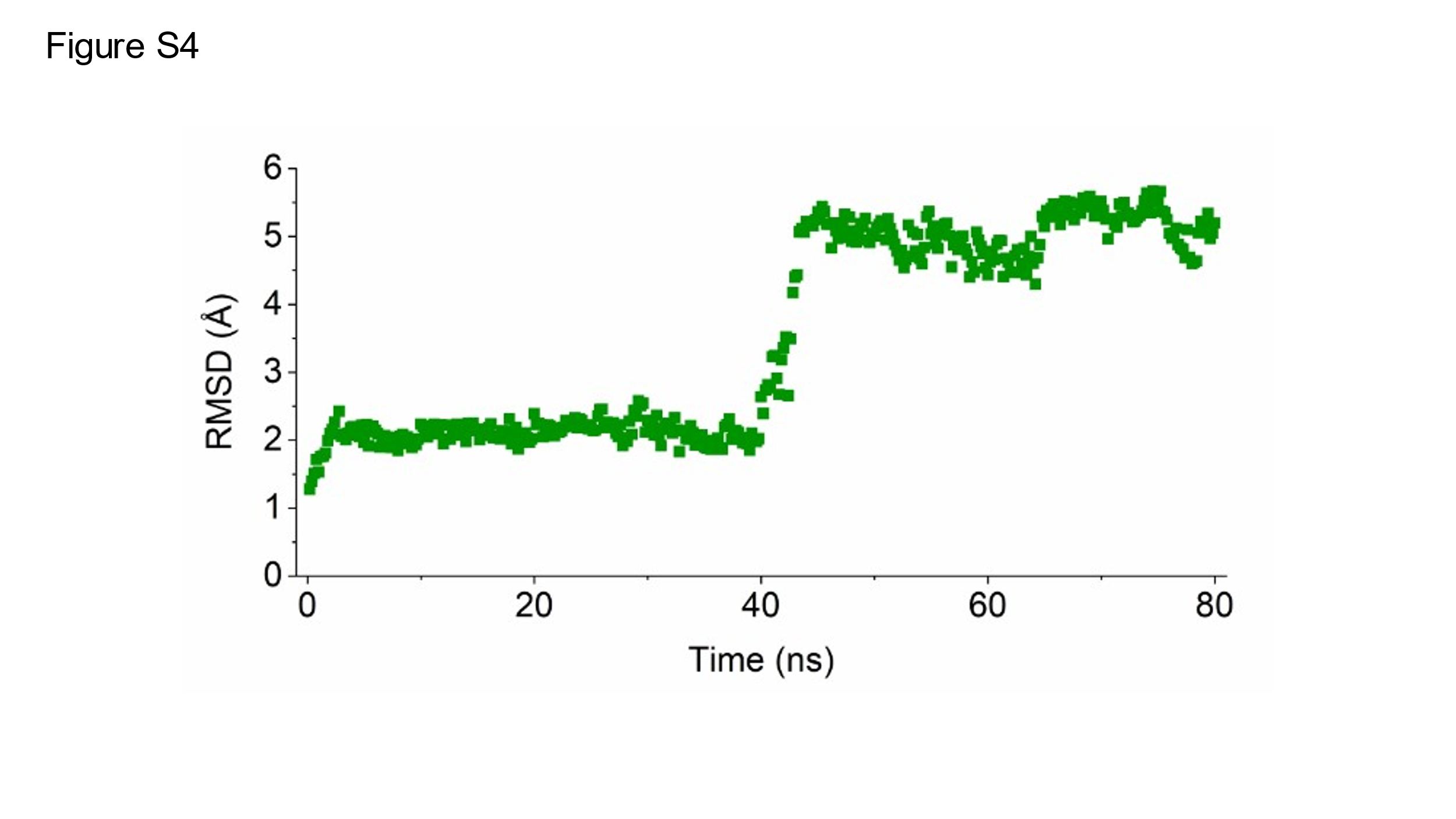

Figure S5
